# Direct identification of microorganisms from thioglycolate broth by MALDI-TOF MS

**DOI:** 10.1371/journal.pone.0185229

**Published:** 2017-09-21

**Authors:** Jorge Arca-Suárez, Fátima Galán-Sánchez, Pilar Marin-Casanova, Manuel A. Rodríguez-Iglesias

**Affiliations:** 1 Laboratorio de Microbiología, Hospital Universitario Puerta del Mar, Cádiz, Spain; 2 Área de Microbiología, Universidad de Cádiz, Cádiz, Spain; Universita degli Studi di Parma, ITALY

## Abstract

We developed an easy MALDI-TOF MS-based assay to identify microorganisms directly from thioglycolate broth. A total of 101 positive thioglycolate broths inoculated with 15 different kinds of samples were evaluated. In 91 samples (90.1%), direct MALDI-TOF MS identifications were the same as those obtained after conventional laboratory procedures including subcultures. In 10 samples misidentified by direct processing, yeasts or mixed cultures grew in the thioglycolate subcultures, or high cellular debris hampered a correct analysis. This rapid method can provide a fast, clinically- relevant species-level identification without disturbing the daily workflow in clinical microbiology laboratories.

## Introduction

It is generally accepted that outcomes of infected patients rely on early and adequate targeted therapy. In this situation, a rapid microbiological diagnosis is crucial for optimal management of infections [1]. Matrix-Assisted Laser Desorption/Ionization Time of Flight Mass Spectrometry (MALDI-TOF MS) has emerged as a reference tool in clinical microbiology laboratories, overcoming older identification methods both in speed and accuracy [2]. Originally devised for use with pure agar cultures, the system has successfully identified bacteria from different crude samples, such as urine, blood cultures or CSF without modifications to the detection algorithms or reference databases [3,4]. Unfortunately, biological debris and clinical samples that harbour low bacterial loads hamper direct analysis. Thus, conventional cultures on agar plates and semisolid nutritive broths remain the gold standard for achieving bacterial growth and completing the microbiological analysis [5]. Nutritive broths like thioglycolate offer a satisfactory recovery rate of microorganisms [6] but require incubation and subsequent subculture in agar before mass spectrometry can be performed. This represents a considerable delay in bacteriological identification and can result in severe clinical and epidemiological consequences. We recently developed a mass-spectrometry-based assay to identify microorganisms directly from thioglycolate broths that is easily implemented and can reduce turnaround times up to 72 hours if slowly growing pathogens are present.

## Materials and methods

In this study, microorganisms are involved, obtained from routinary processing of the samples, and data were analyzed anonymously. We think that our study does not require an ethics statement. The study was performed prospectively between July and October 2016 in the Puerta del Mar University Hospital, a tertiary hospital in Cádiz, Spain. All samples routinely inoculated into thioglycolate broth were evaluated and only those that presented bacterial growth, which was detected visually, were included as positive samples. Ten broths inoculated with clinical samples that did not yield bacterial growth after 14-day incubation, and ten sterile non-inoculated broths were included as negative controls. All broths were incubated a minimum of 14 days before being ruled out.

Positive samples were processed in two ways: on the one hand, broths were inoculated in agar plates and studied by conventional laboratory procedures, checking daily up to 72 hours for the presence of bacterial growth to perform MALDI-TOF MS (Bruker Daltonics) on the grown colonies. Presence of anaerobic bacteria was investigated in all the broths included in the study. On the other hand, MALDI-TOF MS was performed directly on the broth.

Direct processing wasperformed as follows (dx.doi.org/10.17504/protocols.io.hneb5be): once turbidity was detected, 1.5 mL were extracted from the broth with a Pasteur pipette, mixed with 50 μL of 10% Triton and centrifuged (16400g/2 minutes). Then, after the supernatant was discarded, the pellet was suspended in 500 μL of distilled water and re-centrifuged in the same conditions. The resulting pellet was suspended in 50 μL of absolute ethanol. This final suspension was briefly vortexed and 1 μL was finally spotted onto a MALDI-TOF MS target plate to extract proteins with 1μL of 100% formic acid. The resulting spot was air-dried and then overlaid with 1μL of a satured matrix solution of α-cyano-4-hydroxycinnamic acid (HCCA) in organic solvent (50% acetonitrile and 2,5% trifluoroacetic acid) for mass spectrometry. Each test was conducted in triplicate, and only the match with the highest score was recorded for each sample. The Microflex LT system (Bruker Daltonics) was used for analysis of protein extracts, following the manufacturer’s recommendations for calibration. The spectra were initally recorded at 60 Hz, reducing this frequency to minimum to manually acquire the spots if no spectra were recorded after the first test. Resulting spectra were analysed automatically with MALDI Biotyper 3.0 (Bruker Daltonics). The peak list generated was matched against reference libraries, with the integrated pattern-matching algorithm of the software. MALDI-TOF MS identifications were classified by modifying score values proposed by the manufacturer: a score ≥1.7 indicated species identification and a score ≥1.4 indicated genus identification. In addition, whenever the identification score was between 1.7 and ≥1.4, species identification was set as acceptable if the first three successive identification proposals provided by MALDI Biotyper 3.0 matched the same microorganism (3/3). Identifications with scores lower than 1.4 were considered as unreliable. Results obtained directly were compared with those obtained by standard laboratory procedures after performing MALDI-TOF MS on the colonies obtained in pure agar cultures.

## Results and discussion

A total of 101 thioglycolate broths in which bacterial growth was detected visually were evaluated as positive samples. These broths had been inoculated with 15 different types of samples: 25 wound exudates, 15 liquid from endoscope sterility controls, 12 cerebrospinal fluids (CSF), 10 ulcer exudates, 9 abscesses, 8 peritoneal fluids, 7 skin biopsies, 5 central catheters, 2 conjunctival exudates, 2 pleural liquids, 2 biles, 1 corneal cap, 1 umbilical exudate, 1 placenta, and 1 aortic valve. Number of microorganisms, species, and distribution of MALDI-TOF MS scores are shown in Table 1. Ninety-one (90,1%) samples were successfully identified, and MALDI-TOF MS analysis performed on grown colonies in agar plates after subcultures and conventional processing revealed a 100% species-level concordance. The average scores were 2.166 for Gram-negatives, 1.811 for Gram-positives, and 1.722 for anaerobes. In 10 samples misidentified by direct processing, yeasts or polymicrobial flora grew in the thioglycolate subcultures, or high cellular debris hampered a correct analysis (Table 2). In 7 of them no peaks were detected after three trials, while 3 did not reach minimum identification criteria and were considered as unreliable. In the 20 broths used as controls, no peaks were detected.

**Table 1:** MALDI-TOF MS score distribution of the microorganisms successfully identified with the direct thyoglicolate method.

MALDI-TOF MS Identification	MALDI -TOF MS SCORE RANGES	
	≥1.700	1.699–1.4
**Gram-negatives (n = 46)**		
*Enterobacteraerogenes*(n = 2)	2	-
*Enterobactercloacae* (n = 6)	6	-
*Escherichiacoli* (n = 8)	8	-
*Haemophilusinfluenzae* (n = 1)	1	-
*Klebsiellapneumoniae* (n = 3)	3	-
*Morganellamorganii*(n = 4)	4	-
*Proteus mirabilis* (n = 5)	5	-
*Pseudomonas aeruginosa* (n = 11)	11	-
*Pseudomonas mosseilii*(n = 2)	2	-
*Salmonella* sp. (n = 1)	1	-
*Caulobacter*sp. (n = 1)	1	-
*Neisseriaflavescens* (n = 1)	1	-
*Rhizobiumradiobacter* (n = 1)	1	-
**Gram-positives (n = 34)**		
*Enterococcusfaecalis*(n = 6)	6	-
*Enterococcusfaecium* (n = 1)	1	-
*Staphylococcusaureus* (n = 3)	2	1
Coagulase-Negative*Staphylococcus*(n = 11)	7	4
*Streptococcus* spp. (n = 11)	10	1
*Bacillus cereus* (n = 2)	2	-
**Anaerobes (n = 10)**		
*Bacteroides fragilis* (n = 1)	1	-
*Clostridium paraputrificum* (n = 1)	1	-
*Clostridiumtertium*(n = 1)	1	-
*Prevotellabivia* (n = 1)	1	-
*Propionibacteriumacnes* (n = 6)	2	4
**Yeasts (n = 1)**		
*Candidaalbicans* (n = 1)	-	1
**Total (n = 91)**	**80/91 (87,9%)**	**11/91(12,1%)**

**Table 2:** MALDI-TOF MS results and final identification obtained by conventional processing in all misidentified broths.

Sample	Direct MALDI-TOF MS processing			Conventional processing
	ID	Score	Result	
Abdominal abscess	*Mycobacterium scrofulaceum* (1) ^b^	1.206	No reliable identification	*Staphylococcus epidermidis*
Abdominal abscess	-	<0	No peaks found	Polymicrobial flora^a^
Abdominal abscess	-	<0	No peaks found	Polymicrobial flora^a^
Abdominal abscess	-	<0	No peaks found	*Candida albicans*
Central catheter	*Shewanella algae* (1) ^b^	1.335	No reliable identification	*Pseudomonas aeruginosa*
Cervical abscess	-	<0	No peaksfound	Polymicrobialflora^a^
Cerebrospinal fluid	-	<0	No peaks found	*Candida parapsilopsis*
Facial abscess	*Paenibacillus*sp. (1) ^b^	1.171	No reliable identification	*Staphylococcus epidermidis*
Wound exudate	-	<0	No peaks found	Polymicrobial flora^a^
Skin biopsy	-	<0	No peaks found	*Candida albicans*

^a^ More than 4 microorganisms were isolated in agar plates after processing the broth by conventional laboratory procedures.

^b^(N°): Number of consecutive identification proposals provided by MALDI Biotyper 3.0 for the microorganisms with unreliable identifications by direct MALDI-TOF MS (<1.4).

To our knowledge, this is the first proposed assay for direct identification from a nutritive broth using MALDI-TOF MS. Since protein extraction is based on triton, water washes and ethanol, it does not require specific infrastructure nor trained personnel for performance, costs are low and results are obtained rapidly. Compared to conventional processing, this method can reduce turnaround time for at least one working day, even more if slow-growing pathogens are present, providing accurate species-level identification within 30 minutes. Since these microorganisms frequently require 48-hour minimum incubation periods, a guided therapy can be established earlier thereby potentially improving patients clinical outcome. It also offers a remarkably high throughput for clinical CSF or peritoneal fluid specimens, which are often involved in life-threatening infections and frequently carry minimal bacterial loads, yielding growth exclusively in highly nutritive media.

Regarding bacterial identifications, we consider that the results obtained with anaerobic bacteria are clinically relevant, since microorganisms like *Propionibacterium acnes* present a slow growth rate and are highly prevalent in infections with significant morbidity such as bone and joint infections or surgical wound infections[7]. Although scores obtained with anaerobic bacteria were persistently lower than in other microorganisms, all isolates were correctly identified. Concerning other microorganisms, it is noteworthy that 80 isolates (46 Gram-negatives and 34 Gram-positives) were successfully identified by direct MALDI-TOF MS, showing a 100% concordance at species level with conventional laboratory procedures. Although a slight score decrease was observed in Gram-positives, this was not surprising since it is already described that MALDI-TOF MS identification reliability is higher for Gram-negatives [8].

It must be noted that since direct MALDI-TOF MS protocols have emerged in clinical microbiology laboratories, primarily focused on direct identification of microorganisms in blood cultures and urines, cut-off points to be considered have started to be a source of controversy [9]. Current score values accepted (>2 for species identification and >1.7 for genus identification) guarantee excellent identification specificity. Nevertheless, several investigators have suggested that using less stringent MALDI–TOF MS cut-off criteria for direct identification protocols increases sensitivity while maintaining specificity [10–12]. According to observations by Moussaoui et al., when at least four successive species proposals (4/4) with first scores with >1.4 were obtained directly from blood cultures, species identification was never false in their series [13]. Moreover, La Scola and Raoult evaluated a direct protocol with a set of 562 clinical samples applying a very significant reduction in cut-off values (≥1.2, 4/4) and observed a significant improvement in correct identification rates [14]. The results of the present study corroborate the advantage of lowering the cut-off scores applied, since by adjustment of cut-off scores with our criteria, we successfully identified at species level all microorganisms. However, it is noteworthy that if cut-off values suggested by manufacturer were applied, 80 microorganisms (87,9%) would have been correctly identified at genus-level (score ≥1.7), what demonstrates that this methodology can provide reliable identifications using more strict cut-off values.

Among the limitations observed in this work, ten thioglycolathe broths (9,9%) were misidentified, although all of them presented high turbidity and the proteomic profiles for the microorganisms finally identified by conventional processing were present in databases. Most of these broths were inoculated with abscesses. The results of this work show that this method presents a lower performance for these samples, since only three out of nine abscesses yielded successful identifications. We hypothesize that these biological materials present in all misidentified broths could theoretically account for wrong identifications, since these contaminants interfere with spectrum and peak matching [15]. This fact could explain the unexpected unreliable identifications obtained in three of the misidentified broths, since monomicrobial growth was obtained after conventional laboratory procedures. Otherwise, in the seven remaining misidentified samples results show that no peaks can be matched against reference libraries when we analyze broths inoculated with abscesses that also harbour polymicrobial flora, in which spectral overlapping frequently occurs [16–18], or yeasts, that present a robust cell wall that hampers the liberation of an adequate amount of internal proteins needed for a direct analysis [19]. Regarding yeasts, although we successfully identified one *Candida albicans*in this work, Spanu et al. proposed some preprocessing approaches to isolate and purify fungal proteins to perform direct MALDI-TOF on clinical samples, though these modifications are cumbersome and increase turnaround times, abrogating the main advantage of this method [20].

To summarize, the MALDI-TOF MS protocol we have developed, is a low-cost eligible methodology to directly identify microorganisms in thioglycolate broths, providing a fast, clinically- relevant species-level identification without disturbing the daily workflow in clinical microbiology laboratories.
